# Cytomegalovirus (CMV) Reactivation and CMV-Specific Cell-Mediated Immunity After Chimeric Antigen Receptor T-Cell Therapy

**DOI:** 10.1093/cid/ciad708

**Published:** 2023-11-17

**Authors:** Eleftheria Kampouri, Sarah S Ibrahimi, Hu Xie, Elizabeth R Wong, Jessica B Hecht, Mandeep K Sekhon, Alythia Vo, Terry L Stevens-Ayers, Damian J Green, Jordan Gauthier, David G Maloney, Ailyn Perez, Keith R Jerome, Wendy M Leisenring, Michael J Boeckh, Joshua A Hill

**Affiliations:** Vaccine and Infectious Disease Division, Fred Hutchinson Cancer Center, Seattle, Washington, USA; Vaccine and Infectious Disease Division, Fred Hutchinson Cancer Center, Seattle, Washington, USA; Clinical Research Division, Fred Hutchinson Cancer Center, Seattle, Washington, USA; Vaccine and Infectious Disease Division, Fred Hutchinson Cancer Center, Seattle, Washington, USA; Vaccine and Infectious Disease Division, Fred Hutchinson Cancer Center, Seattle, Washington, USA; Vaccine and Infectious Disease Division, Fred Hutchinson Cancer Center, Seattle, Washington, USA; Vaccine and Infectious Disease Division, Fred Hutchinson Cancer Center, Seattle, Washington, USA; Vaccine and Infectious Disease Division, Fred Hutchinson Cancer Center, Seattle, Washington, USA; Translational Science and Therapeutics Division, Fred Hutchinson Cancer Center, Seattle, Washington, USA; Department of Medicine, University of Washington, Seattle, Washington, USA; Clinical Research Division, Fred Hutchinson Cancer Center, Seattle, Washington, USA; Department of Medicine, University of Washington, Seattle, Washington, USA; Clinical Research Division, Fred Hutchinson Cancer Center, Seattle, Washington, USA; Department of Medicine, University of Washington, Seattle, Washington, USA; Department of Laboratory Medicine and Pathology, University of Washington, Seattle, Washington, USA; Vaccine and Infectious Disease Division, Fred Hutchinson Cancer Center, Seattle, Washington, USA; Department of Laboratory Medicine and Pathology, University of Washington, Seattle, Washington, USA; Clinical Research Division, Fred Hutchinson Cancer Center, Seattle, Washington, USA; Public Health Sciences Division, Fred Hutchinson Cancer Center, Seattle, Washington, USA; Vaccine and Infectious Disease Division, Fred Hutchinson Cancer Center, Seattle, Washington, USA; Clinical Research Division, Fred Hutchinson Cancer Center, Seattle, Washington, USA; Department of Medicine, University of Washington, Seattle, Washington, USA; Vaccine and Infectious Disease Division, Fred Hutchinson Cancer Center, Seattle, Washington, USA; Clinical Research Division, Fred Hutchinson Cancer Center, Seattle, Washington, USA; Department of Medicine, University of Washington, Seattle, Washington, USA

**Keywords:** chimeric antigen receptor, immunotherapy, CAR, cytomegalovirus, CMV

## Abstract

**Background:**

The epidemiology of cytomegalovirus (CMV) after chimeric antigen receptor–modified T-cell immunotherapy (CARTx) is poorly understood owing to a lack of routine surveillance.

**Methods:**

We prospectively enrolled 72 adult CMV-seropositive CD19-, CD20-, or BCMA-targeted CARTx recipients and tested plasma samples for CMV before and weekly up to 12 weeks after CARTx. We assessed CMV-specific cell-mediated immunity (CMV-CMI) before and 2 and 4 weeks after CARTx, using an interferon γ release assay to quantify T-cell responses to IE-1 and pp65. We tested pre-CARTx samples to calculate a risk score for cytopenias and infection (CAR-HEMATOTOX). We used Cox regression to evaluate CMV risk factors and evaluated the predictive performance of CMV-CMI for CMV reactivation in receiver operator characteristic curves.

**Results:**

CMV was detected in 1 patient (1.4%) before and in 18 (25%) after CARTx, for a cumulative incidence of 27% (95% confidence interval, 16.8–38.2). The median CMV viral load (interquartile range) was 127 (interquartile range, 61–276) IU/mL, with no end-organ disease observed; 5 patients received preemptive therapy based on clinical results. CMV-CMI values reached a nadir 2 weeks after infusion and recovered to baseline levels by week 4. In adjusted models, BCMA-CARTx (vs CD19/CD20) and corticosteroid use for >3 days were significantly associated with CMV reactivation, and possible associations were detected for lower week 2 CMV-CMI and more prior antitumor regimens. The cumulative incidence of CMV reactivation almost doubled when stratified by BCMA-CARTx target and use of corticosteroids for >3 days (46% and 49%, respectively).

**Conclusions:**

CMV testing could be considered between 2 and 6 weeks in high-risk CARTx recipients.

## Teaser

Cytomegalovirus (CMV) reactivation occurred in 27% of CMV-seropositive chimeric antigen receptor–modified T-cell immunotherapy (CARTx) recipients within 12 weeks. CMV reactivation was almost twice as high after BCMA- versus CD19-CARTx and in patients receiving >3 days of corticosteroids.

## Graphical Abstract

**Graphical Abstract:**
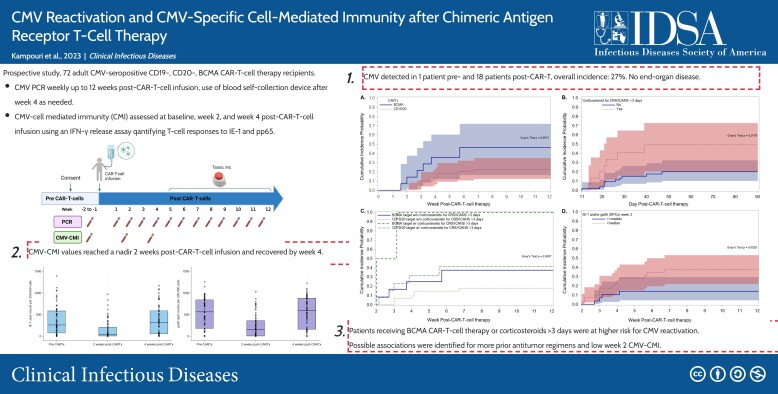
This graphical abstract is also available at Tidbit: https://tidbitapp.io/tidbits/cmv-reactivation-and-cmv-specific-cell-mediated-immunity-after-chimeric-antigen-receptor-t-cell-therapy

Chimeric antigen receptor (CAR)–modified T-cell immunotherapy (CARTx) has revolutionized the management of B-cell and plasma cell malignancies, including B-cell acute lymphoblastic leukemia (ALL) [1, 2], non-Hodgkin lymphoma [3–7], and multiple myeloma [8–10]. However, CARTx comes at the price of unique toxicities, such as cytokine release syndrome (CRS) and immune effector cell–associated neurotoxicity syndrome (ICANS), often requiring immunosuppressive treatment with corticosteroids and/or interleukin 6 inhibitors [11, 12]. The profound immune dysregulation associated with these toxicities, their management, and immune effector cell–associated hematotoxicity, along with preexisting immune deficits, increase the infection risk in these patients. As a result, infections are frequent in the first month after CARTx and are the key determinant of non–relapse mortality [13–18].

Cytomegalovirus (CMV) remains a major pathogen in immunocompromised hosts. CMV frequently reactivates after allogeneic hematopoietic cell transplantation (HCT) and is associated with increased mortality and adverse outcomes [19, 20]. Little is known about the epidemiology of CMV reactivation after CARTx. As such, preventive strategies vary widely, if implemented at all, and are based on expert opinion or extrapolated from autologous HCT approaches [21, 22]. CMV detection is reported in 17%–56% of CD19-CARTx recipients, but prior studies were limited by a retrospective design, small sample size, and/or variable testing frequency and duration, precluding a comprehensive assessment of CMV kinetics, especially beyond the first 4 weeks [18, 23–25].

CMV-specific cell-mediated immunity (CMV-CMI) is key to controlling CMV replication, and CMV-CMI metrics can predict CMV risk after solid organ transplantation [26] and HCT [27]. Because prior antitumor therapies, CARTx-related toxic effects, and resulting corticosteroid use lead to depletion and slow recovery of CD4^+^ and CD8^+^ T cells after CARTx [15, 16], CMV-CMI may be durably impaired, and its measurement may be a valuable tool for post-CARTx CMV risk stratification. The CAR-HEMATOTOX score, developed in recent years, can predict hematotoxicity [28] and infections [29] after CARTx based on readily available biomarkers. Assessing the utility of evolving risk-stratification tools may provide insights to assist individualized clinical decision making. In the current study, we prospectively assessed the incidence, kinetics and risk factors for CMV reactivation in a cohort of CD19-, CD20-, and BCMA-CARTx recipients with weekly testing for up to 12 weeks, incorporating evaluations of CMV-CMI and CAR-HEMATOTOX scores.

## METHODS

### Participants

We prospectively enrolled sequential CMV-seropositive adults planning to receive commercial or investigational CARTx targeting CD19, CD20 or BCMA for non-Hodgkin lymphoma, B-ALL, chronic lymphocytic leukemia (CLL), or multiple myeloma from 1 August 2021 through 17 November 2022 at the Fred Hutchinson Cancer Center in Seattle, Washington (USA). CMV antibody testing was performed routinely in all patients before CARTx. The study was approved by the Fred Hutchinson Cancer Center Institutional Review Board. All participants provided written informed consent.

### Cellular Therapy and Supportive Care

The cellular therapy protocols are summarized in Supplementary Table 1. CRS and ICANS were graded according to the American Society for Transplantation and Cellular Therapy consensus guidelines [12]. Treatment with tocilizumab and/or corticosteroids was considered for patients with grade ≥2 CRS and/or ICANS. Additional therapies may have been used for refractory cases or administered prophylactically as part of a trial (Table 1). Independent of the current study, limited CMV monitoring was recommended according to institutional guidelines (Supplementary Materials). Preemptive treatment was recommended for patients with CMV viral loads ≥150 IU/mL.

**Table 1.** Demographic and Clinical Characteristics of the Cohort

Characteristic	Patients, No. (%)^a^		
	Total (n = 72^b^)	CMV Reactivation (n = 18^c^)	No CMV Reactivation (n = 54)
Follow-up, median (IQR), d	81 (34–84)	62 (34–84)	81 (34–83)
Baseline characteristics			
Age, median (IQR), y	64 (56–70)	62 (52–74)	65 (57–70)
Female sex	31 (43.1)	7 (38.9)	24 (44.4)
Race^d^			
Asian	5 (6.9)	3 (16.7)	2 (3.7)
Black	6 (8.3)	1 (5.6)	5 (9.3)
Native Hawaiian/other Pacific Islander	1 (1.4)	0	1 (1.9)
American Indian/Alaska Native	2 (2.8)	0	2 (3.7)
White	58 (80.6)	14 (77.8)	44 (81.5)
Ethnicity^d^			
Hispanic or Latino	7 (9.7)	1 (5.6)	6 (11.1)
Not Hispanic or Latino	65 (90.3)	17 (94.4)	48 (88.9)
Underlying disease			
Non-Hodgkin lymphoma	52 (72.2)	11 (61.1)	41 (75.9)
ALL	3 (4.2)	0	3 (5.6)
CLL	3 (4.2)	1 (5.6)	2 (3.7)
Multiple myeloma	14 (19.4)	6 (33.3)	8 (14.8)
Prior treatments			
Prior antitumor regimens, median (IQR), no.	4 (3–6)	5 (3–9)	4 (3–5)
>6 Antitumor regimens (upper quartile)	11 (15.3)	6 (33.3)	5 (9.3)
Prior HCT, any	24 (33.3)	5 (27.8)	19 (35.2)
Prior allogeneic HCT^e^	6 (8.3)	3 (16.7)	3 (5.6)
Prior HCT within 1 y^f^	4 (5.6)	0	4 (7.4)
Time since HCT, median (IQR), y	4.2 (1.4, 5.9)	5.2 (4.2, 7.7)	3 (1.2, 5.8)
Prior CARTx	4 (5.6)	0	4 (7.4)
Antibody-based therapy within 6 m^g^	43 (59.7)	10 (55.6)	33 (61.1)
Combination with targeted therapies			
Bruton kinase inhibitors^h^	13 (18.1)	3 (16.7)	10 (18.5)
CAR T-cell target			
CD19/CD20^i^	58 (80.6)	12 (66.7)	46 (85.2)
BCMA	14 (19.4)	6 (33.3)	8 (14.8)
CAR T-cell product			
Axicabtagene ciloleucel	15 (20.8)	5 (27.8)	10 (18.5)
Tisagenlecleucel	3 (4.2)	0	3 (5.6)
Lisocabtagene maraleucel	21 (29.2)	3 (16.7)	18 (33.3)
Brexucabtagene autoleucel	7 (9.7)	1 (5.6)	6 (11.1)
Idecabtagene vicleucel	7 (9.7)	3 (16.7)	4 (7.4)
Ciltacabtagene autoleucel	7 (9.7)	3 (16.7)	4 (7.4)
Investigational product	12 (16.7)	3 (16.7)	9 (16.7)
ALC at baseline, median (IQR), cells/μL^j^	690 (400–1080)	710 (500–1240)	670 (320–1010)
Lymphopenia (≤500 cells/μL)	24 (33.3)	5 (27.8)	19 (35.2)
High CAR-HEMATOTOX score^k^	22 (30.6)	5 (27.8)	17 (31.5)
Post-CARTx clinical features			
CRS, any	54 (75)	14 (77.8)	40 (74.1)
Grade 0	18 (25)	4 (22.2)	14 (25.9)
Grade 1	29 (40.3)	7 (38.9)	22 (40.7)
Grade 2	24 (33.3)	7 (38.9)	17 (31.5)
Grade 3	1 (1.4)	0	1 (1.9)
Grade 4	0	0	0
ICANS, any	29 (40.3)	8 (44.4)	21 (38.9)
Grade 0	43 (59.7)	10 (55.6)	33 (61.1)
Grade 1	8 (11.1)	1 (5.6)	7 (13.0)
Grade 2	7 (9.7)	3 (16.7)	4 (7.4)
Grade 3	12 (16.7)	4 (22.2)	8 (14.8)
Grade 4	2 (2.8)	0	2 (3.7)
CRS and/or ICANS grade ≥2	32 (44.4)	11 (61.1)	21 (38.9)
Immunosuppression for CRS/ICANS^l^	31 (43.1)	11 (61.1)	20 (37.0)
Corticosteroids only	5 (6.9)	1 (5.6)	4 (7.4)
Tocilizumab only	1 (1.4)	0	1 (1.9)
Combination therapy^m^	25 (34.7)	10 (55.6)	15 (27.8)
Steroids, any	30 (41.7)	11 (61.1)	19 (35.2)
High corticosteroid exposure^n^	17 (23.6)	7 (38.9)	10 (18.5)
Duration of steroids, mean (SD), d	2.9 (5.5)	4.1 (5.2)	2.4 (5.6)
>3 d of steroids	17 (23.6)	7 (38.9)	10 (18.5)

Abbreviations: ALC, absolute lymphocyte count; ALL, acute lymphoblastic leukemia; CAR, chimeric antigen receptor; CARTx, CAR-modified T-cell immunotherapy; CLL, chronic lymphoblastic leukemia; CRS, cytokine release syndrome; HCT, hematopoietic cell transplantation; ICANS, immune effector cell–associated neurotoxicity syndrome; IQR, interquartile range; SD, standard deviation.

^a^Data represent no. (%) of patients, unless otherwise specified.

^b^Three patients received repeated infusions, for a total of 72 infusions in 69 patients.

^c^One patient had pre–CAR–T-cell infusion CMV reactivation and is not included here.

^d^Race and ethnicity were self-identified.

^e^Four patients received allogeneic and autologous HCT.

^f^All HCTs during the year before CAR–T-cell infusion were autologous.

^g^Monoclonal antibodies targeting B-cells within 6 months, including rituximab (n = 35), blinatumomab (n = 1), obinutuzumab (n = 2), polatuzumab vedotin (n = 11), inotuzumab ozogamicin (n = 1), daratumumab (n = 3), and belantamab (n = 1).

^h^Administered within a month before CAR–T-cell therapy and including acalabrutinib (n = 8), ibrutinib (n = 2), pirtobrutinib (n = 1), and zanubrutinib (n = 2).

^i^CD20-targeted CAR-T cells in 8 patients (CMV reactivation, n = 3; no CMV reactivation, n = 5).

^j^Lowest ALCs between days −14 and −5 (before lymphodepleting chemotherapy).

^k^Data were available data for 59 patents, with high scores defined as scores ≥2.

^l^Excluding prophylactic anakinra as part of a trial in 8 patients (3 of whom required additional treatment for CRS/ICANS).

^m^Corticosteroids and tocilizumab (n = 17); corticosteroids, tocilizumab, and anakinra (n = 6); corticosteroids and anakinra (n = 2). One patient required high-dose corticosteroids, including intrathecal hydrocortisone, anakinra, and cetuximab (attempt to eliminate CAR–T-cell activity).

^n^More than 3 days of dexamethasone at ≥10 mg/d within a 7-day period (and/or ≥1 dose of methylprednisolone at ≥1 g/d).

### Sample Collection and Testing

We obtained blood samples for isolation of plasma once before lymphodepleting chemotherapy and weekly after CARTx for up to 12 weeks (Supplementary Figure 1). For samples after week 4, participants could use a novel device (Tasso) for home-based self-collection of blood and shipment to our center. We obtained blood for isolation of peripheral blood mononuclear cells (PBMCs) once before lymphodepleting chemotherapy and at weeks 2 and 4 after CARTx; processing is detailed in the Supplementary Materials.

We tested plasma samples for CMV using a laboratory-developed quantitative polymerase chain reaction (PCR) assay with a lower limit of quantitation of 50 IU/mL [30]. If CMV testing was performed for clinical purposes, the Abbott Real Time PCR assay was used, with a limit of quantitation of 50 IU/mL. When results were available from both research and clinical samples within 24 hours, the higher value was used. Results from research testing were not available to clinical teams and did not influence management.

We tested PBMCs for CMV-specific T-cell responses to IE-1 and pp65 antigens using a CMV enzyme-linked immunospot assay (T-SPOT.*CMV*; Oxford Immunotec) (Supplementary Materials). CMV-CMI was determined by counting the number of interferon γ–producing CD4^+^ and CD8^+^ T cells per 250 000 PBMCs (spot counts [SPCs]) after stimulation with CMV antigens IE-1 and pp65 [27]. If the input PBMC count was below the assay's threshold, the result was interpreted as negative. We also generated data for the T-SPOT.*CMV* assay from 10 CMV-seropositive adult volunteers for comparison.

The CAR-HEMATOTOX score was calculated for all participants with available results based on 5 markers obtained before CAR–T-cell infusion: platelet count, absolute neutrophil count, hemoglobin, C-reactive protein, and ferritin [28]. Scores ≥2 were considered high [28].

### Statistical Analyses

We conducted a priori sample size calculations to determine a target enrollment of 70 participants (Supplementary Materials). We calculated the cumulative incidence of CMV reactivation within 12 weeks after CARTx, treating death as a competing risk and comparing curves stratified by baseline variables found to be associated with CMV reactivation using Gray's test. We used box plots to display and compare CMV-CMI values between participants with or without subsequent CMV reactivation, using Wilcoxon rank sum and Wilcoxon signed rank tests as appropriate.

Receiver operating characteristic curves were computed to evaluate the performance of CMV-CMI values at week 2 after CARTx for predicting subsequent CMV reactivation. We used logistic regression to evaluate risk factors for low week 2 CMV-CMI. We used Cox proportional hazards regression to evaluate risk factors for CMV reactivation (variables in Table 1). Given the limited number of CMV reactivation events, we built multivariable models in which CARTx target and corticosteroids were included as key risk factors; additional factors with *P* values <.2 in the univariate model were considered in multivariable models if inclusion modified the effect of corticosteroids by >10% or yielded an adjusted *P* value <.01. Tests were 2 sided with a significance level of .05. Analyses were performed using Stata/SE (version 17.0; StataCorp) or SAS (version 9.4 TS1M3; SAS Institute) software.

## RESULTS

### Patient and Treatment Characteristics

**Figure 1.**
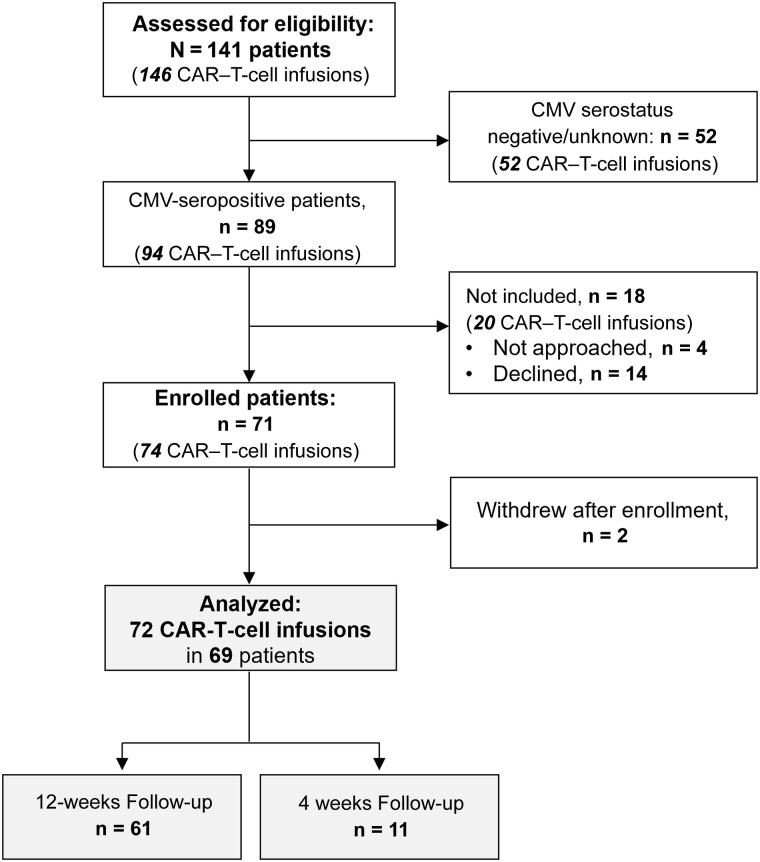
CONSORT flow diagram. Between 1 August 2021 and 17 November 2022, a total of 146 chimeric antigen receptor (CAR)–T-cell infusions were administered in 141 patients with B-cell hematologic cancer, excluding 6 patients who did not sign general consent and were excluded. Among 89 cytomegalovirus (CMV)–seropositive patients, 14 declined, 4 were not approached, and 2 withdrew after enrollment, leading to a total of 72 CAR–T-cell infusions in 69 patients analyzed; consent to follow-up for 12 weeks was obtained in 61 infusion events, and consent to follow-up for up to 4 weeks in 11 infusion events.

During the study period, 141 patients received 146 CAR–T-cell infusions for hematologic cancer; 89 were CMV seropositive and considered for inclusion. We enrolled 71 patients, 2 of whom withdrew; 69 patients who received 72 CAR–T-cell infusions were analyzed (Figure 1). There were no notable differences in baseline characteristics between participants and non-participants (Supplementary Table 2). Participants received a median of 4 prior antitumor regimens (interquartile range [IQR], 3–6), and 24 (33.3%) received prior HCT. Fifty-eight patients (80.6%) received CD19- or CD20-CARTx for non-Hodgkin lymphoma (n = 52 [72.2%]), ALL (n = 3 [4.2%]), or CLL (n = 3 [4.2%]); BCMA-CARTx was administered in 14 patients (19.4%) with multiple myeloma. CRS and/or ICANS grade ≥2 occurred in 32 (44.4%) participants, and 30 (41.7%) received corticosteroids (Table 1). Thirteen patients experienced relapse, and 2 died during study follow-up. Home-based self-collection of blood samples was performed beyond week 4 in 37 patients and was well tolerated.

### Incidence and Characteristics of CMV Reactivation

**Figure 2.**
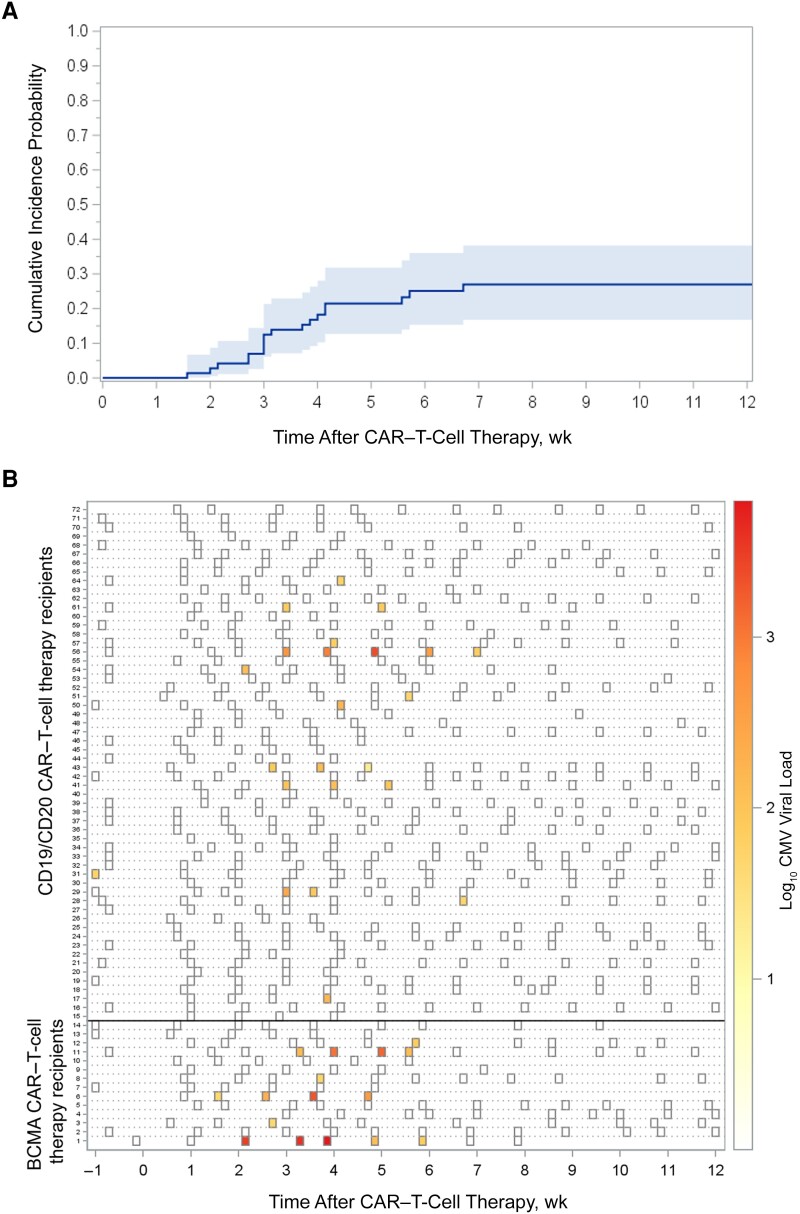
Cumulative incidence and kinetics of cytomegalovirus (CMV) reactivation within 12 weeks after chimeric antigen receptor (CAR) T-cell infusion. *A,* Cumulative incidence curve of any CMV reactivation within 12 weeks: 27% (95% confidence interval; 17%–38% (depicted by shading). *B,* Heat map of CMV reactivation kinetics. Each row represents a patient, and each square a plasma sample. The intensity of color represents the viral load (negative samples are depicted as white). BCMA-CARTx recipients are depicted at the bottom, and CD19/CD20 CARTx recipients at the top of the heat map.

Participants were followed up for a median of 81 (IQR, 34–84) days and had a median of 11 (IQR, 6–13) tests. CMV reactivation (60 IU/mL) was detected in 1 participant (1.4%) before CARTx, without subsequent positive test results. After CARTx, CMV reactivation occurred in 18 participants (25%) at a median of 22 (IQR, 19–29) days, for a cumulative incidence of 22% (95% confidence interval (CI), 13%–32%) by week 4% and 27% (95% CI, 17%–38%) by week 12 (Figure 2*A*). All post-CARTx reactivations occurred within 2 to 6 weeks, except in 2 participants with CMV reactivation on days 11 and 47.

The kinetics of CMV reactivation are depicted in Figure 2*B*. The median peak viral load was 127 (IQR, 61–276) IU/mL; 7 patients (10%) had a viral load above the preemptive treatment threshold (≥150 IU/mL). CMV clinical testing was performed in 23 patients with positive in 9 patients (41% of those tested); 5 were preemptively treated with valganciclovir (Supplementary Materials). No patients had CMV end-organ disease diagnosed (Table 2).

**Table 2.** Virologic Characteristics of CMV Reactivation

Characteristics	Patients, No. (%) (n = 72)^a^
No. of tests per patient, median (IQR)	11 (6–13)
Patients with CMV reactivation after CAR–T-cell therapy	18 (25)
No. of tests among patients with ≥1 positive test result, median (IQR)	
Total no. of tests	10 (8–13)
No. of positive test results	1 (1–3)
Proportion of positive results per patient, median (IQR), %	20 (9–25)
VL	
First positive VL, median (IQR), IU/mL^b^	83 (53–164)
Peak VL, median (IQR), IU/mL^b^	127 (61–276)
Peak VL ≥1000 IU/mL	4 (5.6)
CMV VL above treatment threshold (≥150 IU/mL)	7 (9.7)
Time to test in days among patients with a positive test result, median (IQR)	
Time to first positive result	22 (19–29)
Time to peak VL	28 (25–34)
Clinical characteristics	
CMV detected with clinical testing	9 (12.5)
End-organ disease	0
Antiviral therapy	5 (6.9)

Abbreviations: CMV, cytomegalovirus; IQR, interquartile range; VL, viral load.

^a^Data represent no. (%) of participants unless otherwise specified.

^b^Among patients with CMV reactivation.

### CMV-CMI Results

**Figure 3.**
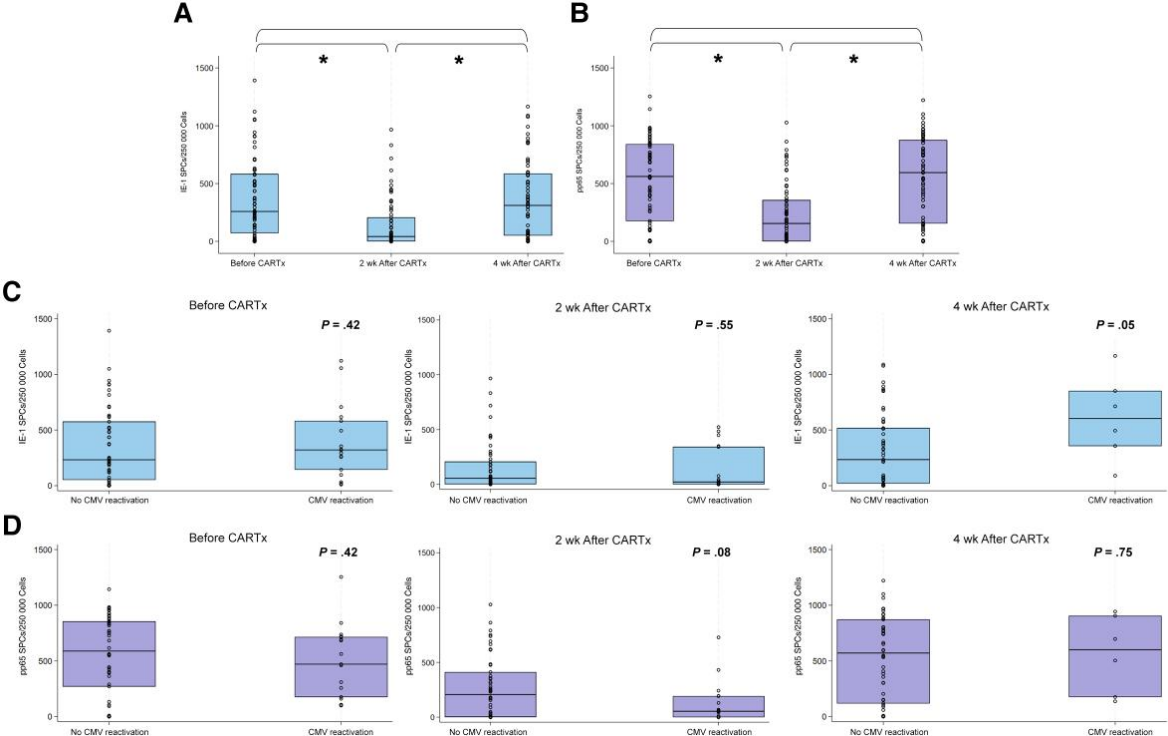
Comparison of cytomegalovirus (CMV)–specific cell-mediated immunity (CMV-CMI) (T-cell responses to IE-1 and pp65) at time points before and 2 and 4 weeks after chimeric antigen receptor–modified T-cell immunotherapy (CARTx), overall and among patients with versus without CMV reactivation. *A, B,* Box plots of CMV-CMI at each time point in all participants for IE-1 (*A*) and pp65 (*B*). *C, D,* Box plots of CMV-CMI at each time point stratified by subsequent development of CMV reactivation or not (relative to each time point) for IE-1 (*C*) and pp65 (*D*); patients with CMV reactivation before each time point are not depicted. Horizontal lines and the boxes represent median values and upper and lower quartiles, respectively; circles represent all values. Wilcoxon rank sum and Wilcoxon signed rank tests were used as appropriate. *P < .001. Abbreviation: SPCs, spot counts.

Longitudinal CMV-CMI for pp65 and IE-1 was measured in 69 patients before and at weeks 2 and 4 after CARTx. Seventeen samples had PBMC counts below the assay's threshold in the setting of low ALC (before CARTx, n = 2; week 2 after CARTx, n = 12; week 4, n = 3) and were assigned a CMV-CMI value of 0. Pre–CAR–T-cell infusion CMV-CMI results were comparable to those in 10 nonimmunocompromised CMV-seropositive controls for both antigens (Supplementary Figure 2). At week 2 after CARTx, CMV-CMI was significantly lower compared with baseline and recovered to baseline levels by week 4 (Figure 3). CMV-CMI by CARTx target is shown in Supplementary Figure 3.

At the pre-CARTx and week 4 post-CARTx time points, IE-1 and pp65 were comparable between participants with and those without subsequent CMV reactivation. At week 2, CMV-CMI tended to be lower in patients with subsequent CMV reactivation than in those without, especially for pp65 (Figure 3).

Based on the observed kinetics of CMV-CMI, as well as the timing of CMV reactivation after CARTx, we focused on CMV-CMI metrics at week 2 as a key time point to assess the potential utility of CMV-CMI for CMV risk stratification. A receiver operating characteristic curve analysis at this time point demonstrated modest sensitivity and specificity for pp65 and IE-1 as predictors of subsequent CMV reactivation (Supplementary Figure 4), wherein median values (155 SPCs for pp65 and 42 SPCs for IE-1) were associated with sensitivities of 61% for IE-1 and 72% for pp65 and specificities of 53% for IE-1 and 57% for pp65. These thresholds had high negative predictive values of approximately 80% but low positive predictive values of <50%.

In a regression model evaluating the association of baseline and antecedent variables with week 2 CMV-CMI, we identified significant associations of lower baseline CMV-CMI, corticosteroids for >3 days, and maximum grade of ICANS with lower responses to IE-1; only low baseline CMV-CMI was associated with lower responses to pp65 (Supplementary Table 4). CMV-CMI at weeks 2 and 4 after CARTx, stratified by prior corticosteroid use, is depicted in Supplementary Table 3.

We also assessed how the ALC performed in predicting subsequent CMV reactivation, by comparing the ALC at the pre-CARTx and week 2 time points, using the lowest ALC for each interval. ALCs at both time points were comparable between participants with or without CMV reactivation (Supplementary Figure 5).

### Risk Factors for CMV Reactivation From Adjusted Models

**Figure 4.**
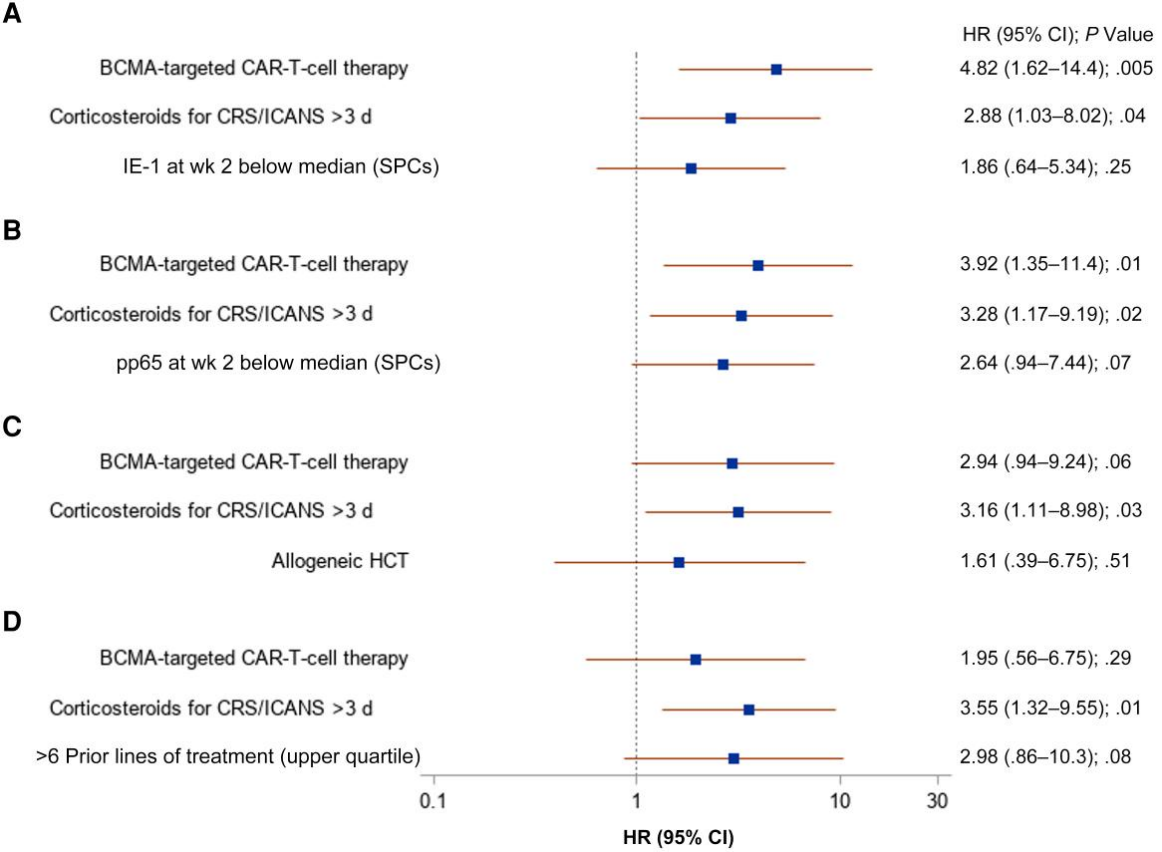
Forest plots of multivariable Cox regression models for cytomegalovirus (CMV) reactivation within 12 weeks after chimeric antigen receptor (CAR) T-cell therapy. Forest plot of 4 models incorporating unique sets of variables. *A, B,* Data on participants with available CMV-specific cell-mediated immunity (CMV-CMI) results (69 infusions in 66 participants). *C, D,* Data on all studied infusions (72 infusions in 69 participants). CMV-CMI and corticosteroids were evaluated as time-dependent variables. Abbreviations: CI, confidence interval; CRS, cytokine release syndrome; HR, hazard ratio; ICANS, immune effector cell–associated neurotoxicity syndrome; SPCs, spot counts.

Potential associations were identified between several variables and subsequent CMV reactivation in univariate models, including more prior antitumor therapy regimens, prior allogeneic HCT, CARTx target, higher CRS or ICANS severity, immunosuppression for CRS or ICANS, and pp65 or IE1 below the median at week 2 (Supplementary Table 5). The CAR-HEMATOTOX score was not associated with CMV reactivation. In multivariable models, BCMA-CARTx and corticosteroid use for >3 days were consistent risk factors for CMV reactivation (Figure 4). Receipt of more prior antitumor regimens, as well as lower CMV-CMI values at week 2, also appeared to be associated with increased CMV reactivation, although these differences did not reach statistical significance.

Of note, BCMA-CARTx recipients received more prior lines of antitumor regimens (median [IQR], 7 [6–11 lines]) compared with CD19/CD20 CARTx recipients (median [IQR], 4 [3–5]; *P* = .001) and more frequent prior HCT (71% vs 24%, respectively; *P* = .001). In models incorporating CRS and/or ICANS grade ≥3 in place of corticosteroids, this variable had lower association with CMV (data not shown). Similar results were obtained when a CMV outcome was defined as >1 positive test result and/or ≥150 IU/mL (n = 10) (Supplementary Table 6 and Supplementary Figure 6).

**Figure 5.**
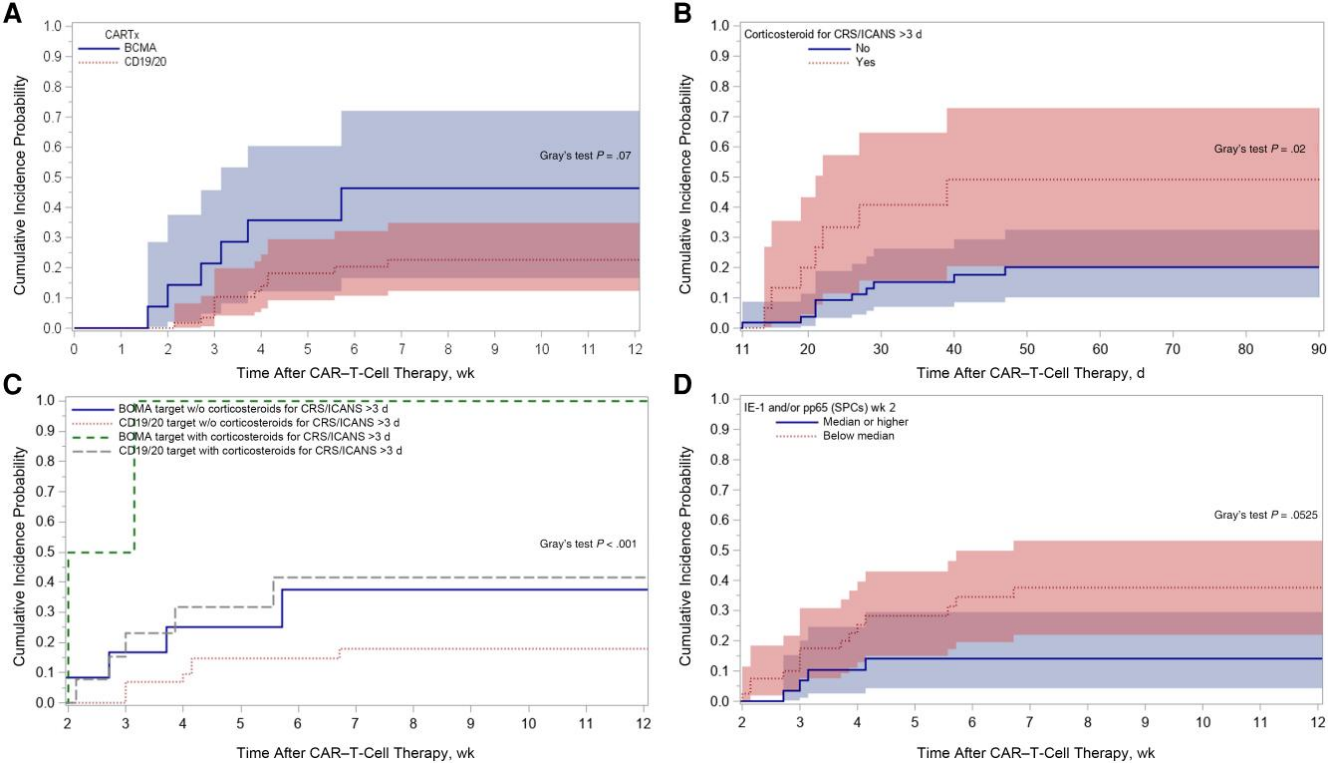
Cumulative incidence of cytomegalovirus (CMV) reactivation by week 12 after chimeric antigen receptor (CAR)–modified T-cell therapy (CARTx), stratified by key clinical characteristics. *A,* Stratification by CAR–T-cell therapy target: BCMA (46% [95% confidence interval (CI), 17%–72%]) versus CD19/CD20 (23% [12%–35%]). *B,* Stratification by receipt of corticosteroids for cytokine release syndrome (CRS)/immune effector cell–associated neurotoxicity syndrome (ICANS) for >3 days: 49% (95% CI, 21%–73%) versus no steroids or steroids for <3 days: 20% (10%–33%). Curves start at day 11, after corticosteroid onset for CRS/ICANS (onset of corticosteroids >3 days by day 11 in all patients but 3, who were excluded from this plot; all CMV events occurred after day 11). *C,* Stratification by CARTx target and receipt of corticosteroids: BCMA with or without (w/o) corticosteroids for >3 days, 100% and 38%, respectively; CD19/20 with or without corticosteroids, 41% and 18%. Curves start at day 11 after corticosteroid onset for CRS/ICANS (onset by day 11 in all but 3 patients, who were excluded from this plot; all CMV events occurred after day 11). *D,* Stratification by presence of low CMV-specific cell-mediated immunity (CMV-CMI) at week 2 (defined as IE-1 and/or pp65 below median): high CMV-CMI, 14% (95% CI, 4%–30%) versus low CMV-CMI: 38% (22%–53%). Curves start at week 2 and include data on 69 patients with available CMV-CMI results. Abbreviation: SPCs, spot counts.

To visualize the utility of risk stratification by key clinical variables identified in the analyses for guiding potential CMV screening, we generated stratified cumulative incidence plots of CMV reactivation by BCMA- versus CD19-CARTx, >3 days of corticosteroids for CRS/ICANS, a composite of BCMA-CARTx and corticosteroid use >3 days, and CMV-CMI values (Figure 5). Within these groups, stratification more than doubled the observed incidence of CMV reactivation and approached 50% in patients receiving corticosteroids for >3 days or BCMA-CARTx. The incidence was lowest in CD19/CD20 CARTx recipients who did not receive corticosteroids (13%). When considering all BCMA-CARTx recipients and/or individuals receiving corticosteroids for any duration, all cases of CMV viremia above the preemptive treatment threshold, and 14 of 18 CMV reactivation events (78%) at any level, were captured.

## DISCUSSION

In this prospective study among CMV-seropositive adult CARTx recipients monitored weekly for CMV for up to 12 weeks, we found an overall cumulative incidence of CMV reactivation of 27%. CMV events were mostly low level and mainly occurred between 2 and 6 weeks after CARTx. End-organ disease did not develop in any participants during the study period. CMV reactivation incidence was more than twice as high in BCMA- compared with CD19-CARTx recipients and in patients receiving corticosteroids for CRS and/or ICANS, especially for >3 days. CMV-CMI reached a nadir 2 weeks after CAR–T-cell infusion and recovered to pre-CARTx levels by 4 weeks after CARTx. In adjusted models, receipt of BCMA-CARTx, corticosteroid use for >3 days, more prior treatment regimens, and lower CMV-CMI at week 2 were associated with CMV reactivation.

Systematic data on CMV reactivation after CD19-CARTx are scarce, and they are completely lacking after BCMA-CARTx. In a few available retrospective studies, the incidence of CMV viremia varied from 17% when a single test was performed 2–3 weeks after infusion [18] to 44%–58% with more frequent testing (median, 5–6 tests) and longer follow-up [23–25]. Despite weekly testing for 12 weeks after CARTx, our estimated incidence of 23% after CD19-CARTx is lower than previously reported in studies with repeated testing, perhaps owing to differences in patient and treatment characteristics.

The CMV incidence was twice as high in BCMA-CARTx recipients as in CD19-CARTx recipients (46% vs 23%, respectively). Although the epidemiology of CMV reactivation after BCMA-CARTx has not been previously assessed to our knowledge, other studies demonstrate a higher incidence of viral infections than in CD19-CARTx recipients [8, 31–34]. Of note, BCMA-CARTx recipients had a higher number of prior antitumor regimens and more frequent prior HCT, as expected based on eligibility for these products only after 4 prior lines of treatment. This may explain the observed difference in CMV risk after BCMA-CARTx, as the significance of BCMA-CARTx was lost in a model accounting for the number of prior treatment regimens.

Reassuringly, we did not observe any CMV end-organ disease, in agreement with most previous studies [18, 23, 25, 35], though CMV end-organ disease has been reported after CARTx [24, 36, 37]. Our relatively small sample size and the use of preemptive therapy preclude an estimation of CMV end-organ disease incidence in this population. CMV viremia in the absence of apparent end-organ disease may also be associated with worse outcomes, similar to what is well-documented after allogeneic HCT, but our study was underpowered for such an analysis [24].

CRS [13, 14] and ICANS [13, 16–18] have been associated with increased risk of infection related to immune dysregulation, endothelial damage, and immunosuppressive treatment required for management. Corticosteroid therapy is also a strong predictor of infection after CD19-CARTx and has an impact on cellular immunity, which is key to controlling CMV [15, 16, 29, 37]. Indeed, corticosteroid use for >3 days for CRS and/or ICANS was a strong risk factor in all models.

Based on an increased understanding of the use of CMV-CMI metrics to predict CMV reactivation and disease after HCT and SOT, we explored their kinetics and utility in this unique patient population using T-SPOT.*CMV*, a commercially available interferon-γ release assay [26, 27]. CMV-CMI reached a nadir at week 2 after CARTx and recovered by week 4. Thresholds of 100 SPCs for pp65 and 50–100 SPCs for IE1 have been shown to predict clinically significant CMV reactivation in HCT recipients in studies using the same assay [27, 38]. In our study, week 2 thresholds of 150 SPCs for pp65 and 40 SPCs for IE-1 (approximate medians) had modest sensitivity and specificity but high negative predictive values for subsequent CMV reactivation. Thus, this test could provide additional guidance as to which patients may not need CMV monitoring or other preventive strategies, but larger studies are needed. Importantly, CMV-CMI values were universally lower in patients receiving corticosteroids, indicating a mechanistic link between corticosteroid use and CMV reactivation risk. We did not find evidence of an association between ALC and CMV reactivation risk, further supporting the relevance of interrogating pathogen-specific T-cell immunity.

Our study has several strengths. To our knowledge, this is the first prospective study of CMV reactivation after CARTx with the most frequent testing and the only systematic study in BCMA-CARTx recipients. We used innovative sampling methods for home-based self-collection of blood samples, an emerging strategy that could be implemented for more robust monitoring of high-risk individuals and for which this is one of few studies in immunocompromised hosts. This is the only study to date reporting longitudinal assessment of CMV-CMI in CARTx recipients. We also assessed the utility of the novel CAR-HEMATOTOX scores in predicting CMV and found no association with CMV reactivation, suggesting that the score is best suited for risk stratification of bacterial infection [28, 29].

Our study also has limitations. The sample size and number of events did not allow for a more complete risk factor analysis, and more data are needed in patients with ALL and CLL owing to low numbers. The preemptive threshold for CMV treatment was based on institutional guidelines for autologous HCT recipients. Our study was underpowered to evaluate how the kinetics of CMV detection affect clinical outcomes. The clinical significance of CMV viremia in the absence of end-organ disease in this patient population requires further analysis in larger studies. Furthermore, due to the low number of CMV events, which were mostly short and low level, we could not fully assess the impact of CMV-CMI on higher viral loads and other virologic kinetics. Finally, we could not analyze the impact of other immunomodulatory agents (eg, tocilizumab) on CMV reactivation risk owing to overlap with corticosteroid use.

In conclusion, our findings suggest that patients receiving BCMA-CARTx, >3 days of corticosteroids, and >6 prior lines of treatment may benefit from CMV surveillance. CMV-CMI at week 2 could help further refine CMV risk assessment and the need for surveillance or prophylactic approaches, although validation in larger cohorts is needed. Additional studies of CMV will be important as novel CARTx approaches are implemented (eg, allogeneic CARTx), because prophylactic treatments could have a role in high-risk patients.

## Supplementary Data

Supplementary materials are available at *Clinical Infectious Diseases* online. Consisting of data provided by the authors to benefit the reader, the posted materials are not copyedited and are the sole responsibility of the authors, so questions or comments should be addressed to the corresponding author.

## Supplementary Material

ciad708_Supplementary_Data

## Notes

***Author contributions.*** E. K. and J. A. H. designed the study. M. J. B. contributed to study design. E. K., H. X., W. M. L., and J. A. H. analyzed and interpreted the data and created the figures. E. K., S. S. I., J. B. H, M. K. S., A. V., and J. A. H. enrolled participants and collected samples and data. E. K., E. R. W., and T. L. S. A. performed and interpreted all cytomegalovirus-specific cell-mediated immunity testing. T. L. S. A., A. P., and K. R. J. supervised the laboratory work. E. K. and J. A. H. prepared the first draft of the manuscript. All authors contributed to the writing and revision of the manuscript and approved the final version.

***Financial support.*** This work was supported by the Swiss National Science Foundation (grant P500PM_202961 to E. K.), the SICPA Foundation (grant to E. K.), Merck (grant to J. A. H.), and Oxford Immunotec (support to J. A. H.; including provision of the T-SPOT.*CMV* tests and T-Cell *Xtend*) and the National Institutes of Health (Cancer Center Support Grant P30 CA015704-47 to the Fred Hutchinson Cancer Center).
