# Supplementary material for: Cytomegalovirus (CMV) Reactivation and CMV-Specific Cell-Mediated Immunity After Chimeric Antigen Receptor T-Cell Therapy
Source: Clin Infect Dis. 2023 Nov 17;78(4):1022–32. doi: 10.1093/cid/ciad708 (PMC11006113; doi:10.1093/cid/ciad708)
Supplement: ciad708_Supplementary_Data [file ciad708_supplementary_data.docx]

**Supplement to CMV Reactivation and CMV-Specific Cell-Mediated Immunity after Chimeric Antigen Receptor T-Cell Therapy**

**METHODS**

**Cellular therapy and supportive care**

All patients received lymphodepleting chemotherapy with fludarabine and cyclophosphamide followed by CAR-T-cell infusion, except for two patients who received bendamustine due to a nationwide shortage of fludarabine. Cellular therapy protocols are detailed in **Table S1**.

Weekly CMV monitoring with quantitative PCR in plasma was recommended in patients receiving high-dose corticosteroids (defined as >3 days of ≥10 mg dexamethasone or equivalent per day within a 7-day period and/or receiving ≥1 dose of methylprednisolone ≥1 gram per day) or >1 dose of tocilizumab and/or other anti-cytokine therapy; testing was recommended for four weeks after the last dose of corticosteroids or anti-cytokine therapy. Preemptive therapy was recommended for any test with CMV ≥150 IU/mL and consisted of valganciclovir 900 mg PO twice a day or ganciclovir 5 mg/kg IV twice a day with foscarnet 90 mg/kg IV twice a day as an alternative (induction dosing), followed by maintenance dosing at least 7 days after CMV viral loads started declining. This approach was extrapolated from preemptive treatment strategies in autologous HCT recipients at our center.

Antimicrobial prophylaxis consisted of acyclovir 800 mg or valacyclovir 500 mg twice a day for herpes simplex or varicella zoster virus seropositive individuals starting on the day of lymphodepletion until a year after CAR–T-cell infusion, levofloxacin 750 mg daily and fluconazole 200 mg daily while the ANC was <500 cells per mm^3^, and trimethoprim 160 mg/sulfamethoxazole 800 mg twice a day for 2 days each week starting after neutrophil recovery until ≥3 months after CAR–T-cell infusion. Posaconazole prophylaxis 300 mg daily (twice daily on first day) is recommended instead of fluconazole if ANC <500 cells per mm^3^ for >20 days or in patients receiving high-dose steroids (defined in main manuscript), >1 dose of tocilizumab or ≥1 doses of other anti-cytokine, until 1 month following the last day of treatment for CRS/ICANS.

**Definitions**

CMV reactivation was defined as detection of CMV DNAemia at any level in blood. Established definitions for CMV end-organ disease were used.[1]

**Sample collection and processing**

Plasma samples were isolated from whole blood collected in EDTA purple top vacutainers (or Tasso+ K2 EDTA tubes for self-collected samples) and were stored at -80°C until batch testing with CMV PCR. Peripheral blood mononuclear cells (PBMCs) were collected in LiHep green top vacutainers for processing to perform the T-SPOT^®^.*CMV* assay; samples were processed within eight hours of draw time or within 32 hours of draw time with the addition of Tcell *Xtend*^®^ (Oxford Immunotec, Abingdon, UK). Whole blood was transferred from vacutainers into 50mL conical tubes and diluted in a 1:1 ratio of blood to room temperature (RT) RPMI 1640 media (Gibco). Blood solution was poured into a Leucosep tube (Grenier Bio-One) prepared with Histopaque (Sigma-Aldrich) and centrifuged (10 min, no brake, 1000g, RT). After centrifugation, the layer above the Leucosep frit was poured into a new 50mL conical tube. RPMI was then added up to the 45mL mark and sample solution was inverted to mix and centrifuged for 10 min with brake at 300g, RT. The sample’s supernatant was then decanted into liquid waste and the pellet resuspended in 45mL RPMI, inverted to mix, and spun once more for 10 min with brake at 300g, RT. Supernatant was decanted and isolated PBMC pellet was resuspended in 1mL of AIM V growth medium (Gibco) at RT.

PBMCs were counted on a K2 cellometer (Nexcelom) using a 20uL AO/PI staining dye (Nexcelom) to 20uL PBMC sample solution and the cell count and viability were recorded. Sample concentration was then diluted in AIM V, left as is or resuspended in a smaller concentration to meet the cell count criteria of 2.0 x 10^6^ to 3.0 x 10^6^ cell concentration required to proceed with the T-SPOT^®^.*CMV* (Oxford Immunotec, Abingdon, UK).

**T-SPOT.CMV ELISPOT assay**

We used a peptide-based enzyme-linked immunospot (ELISPOT) CMV assay (T-SPOT^®^.*CMV*; Oxford Immunotec, Abingdon, UK) to assess CMV-CMI on freshly isolated PBMCs according to the package insert.[2] CMV-CMI was determined by counting the number of IFN-γ-producing CD4+ and CD8+ T cells per 250,000 PBMCs (reported as spot counts [SPC]) after stimulation with CMV antigens IE-1 and pp65.[3] If the input PBMC cell count was below the assay’s threshold, the result was interpreted as negative.

The optimal number of PBMCs to be added to each of the four test wells (a. negative control, b. IE-1, c. pp65, d. positive control) is 250,000. When the cell count ranged from 75,000 to <250,000 cells per well, SPCs for IE-1 and pp65 antigens were standardized by multiplying the result by a correction factor to adjust to the optimal cell count of 250,000 cells per well.[4] If the number of PBMCs was > 250,000 a dilution was performed. If the number of PBMCs was below the assay’s threshold of 75,000 per well (i.e., in case of low absolute lymphocyte count), the result was interpreted as negative. A negative control result of ≤10 SPCs was considered valid and was subtracted from the IE1, pp65 and positive control SPCs per the assay’s protocol. Samples were read on an automated CTL Spot Counting machine (ImmunoSpot, Cleveland, OH).

**Statistical Analyses**

Descriptive statistics were used to summarize demographic and baseline characteristics. We calculated a sample size *a priori* for the primary endpoint of CMV reactivation incidence within 12 weeks post-CARTx. Based on an estimation that ~50% of patients would reactivate CMV, a sample size of 70 participants would provide a 95% confidence interval (CI) for this estimate within +/-12%. Using this sample size, we estimated minimum detectable hazard ratios (HRs) of 2.56–3.11 for a difference in CMV reactivation risk between patients with and without corticosteroid use for CMV reactivation rates of 30%–50% with a 2-sided type I error rate of 0.05 and 80% power. Based on our actual sample size and observations, the minimal detectable HR for CMV reactivation was 2.31 in participants receiving >3 days of corticosteroids versus not based on a power of 80% with a 2-sided type I error rate of 0.05.

We used Cox proportional hazards regression to evaluate risk factors for CMV reactivation. CMV-CMI measurements were integrated as binary variables based on median values and thresholds from ROC analyses. The proportional hazards assumption was tested for all covariates. Model estimates were presented as adjusted hazard ratios (aHR) with 95% CIs.

**Results**

**Incidence and characteristics of CMV reactivation**

CMV reactivation post-CARTx occurred in 18 out of 72 participants, and 7 out of 18 had CMV viral loads above the threshold for preemptive therapy (≥150 IU/mL). Nine out of these patients also had positive clinical testing (monitoring in the context of high-risk criteria per our institutional guideline), and 5 of these 9 had viral load thresholds >150 IU/mL and received preemptive therapy. Among these patients, median peak viral load was 168 IU/mL (IQR, 127–1526 IU/mL), median time to first viral load was 21 days (IQR, 19–28 days), median time to peak viral load was 28 days (IQR, 26–34 days), and median number of positive tests was 2 (IQR, 1–4). Clinical testing was performed in 23 patients in total and was almost uniformly performed in the context of asymptomatic monitoring of high-risk patients. Testing was motivated by clinical manifestations in three patients (abnormal liver tests in 2, one of whom had positive CMV testing, fever and rash in 1 patient with positive CMV testing).

Nine patients tested positive only with research testing and were mostly asymptomatic (fever of unknown origin in one patient with CMV viral load >1000 IU/mL). One additional patient had a single positive CMV test pre-CARTx. Two of these 9 had viral load thresholds >150 IU/mL. Median peak viral load was 77 IU/mL (IQR, 51–127 IU/mL), median time to first viral load was 26 days (IQR, 21–29 days), median time to peak viral load was 27 days (IQR, 25–29 days), and median number of positive tests was 1 (IQR, 1–2).

Treatment was administered in 5 patients based on clinical CMV testing. Two additional patients with CMV viral load above treatment threshold did not have clinical testing and were not treated; one of them had a single positive test (164 IU/mL) and the second patient had CMV viremia for 3 weeks (4 positive tests) and a peak viral load of 2088 IU/mL. The patient had fever of unknown origin. Patients with post-CAR-T CMV reactivation who did not receive treatment (n=13) had a median peak CMV viral load of 77 (IQR, 53–127IU/mL) compared to 1526 IU/mL (IQR, 276–2382 IU/mL) in patients with CMV reactivation receiving treatment (p=0.004), and a median number of positive tests of 1 (IQR, 1–2) compared to 4 (IQR, 2–5) in patients receiving treatment (p=0.03). This demonstrates that the approach to CMV clinical monitoring of selected patients did indeed identify a higher-risk population given that they had higher viral loads and longer duration of viremia despite treatment.

**Table S1.** Details pertaining to the cellular therapy product used for participants on this study.

| **Number of participants** | **NCT Number** | **Product** | **Study phase** | **Target**  **(co-stimulatory domain)** | **Indication** |
| --- | --- | --- | --- | --- | --- |
| 1 | 02631044 | JCAR017  (lisocabtagene maraleucel) | Phase 1 | CD19 (4-1BB) | R/R B-cell NHL |
| 2 | 04245839 | JCAR017  (lisocabtagene maraleucel) | Phase 2 | CD19 (4-1BB) | R/R B-cell NHL |
| 1 | 03331198 | JCAR017  (lisocabtagene maraleucel) | Phase 1/2 | CD19 (4-1BB) | R/R CLL or small lymphocytic lymphoma |
| 1 | 04400591 | lisocabtagene maraleucel  (non conforming) | EAP | CD19 (4-1BB) | R/R B-cell NHL |
| 4 | 03103971 | huJCAR014[5] | Phase 1 | CD19 (4-1BB) | R/R B-cell NHL |
| 1 | 05360238 | MB106 | Phase 1/2 | CD20 (CD28 & 4-1BB) | R/R B-cell NHL or CLL |
| 7 | 03277729 | - | Phase 1/2 | CD20 (CD28 & 4-1BB) | R/R B-cell NHL |
| 1 | 04196491 | bb2121  (idecabtagene vicleucel) | Phase 1 | BCMA (4-1BB) | Multiple myeloma |
| 1 | 04133636 | JNJ-68284528  (ciltacabtagene autoleucel) | Phase 2 | BCMA (4-1BB) | Multiple myeloma |
| **Other Studies** |  |  |  |  |  |
| 8 | 04359784 | anakinra | Phase 2 |  | Prevention of CRS/ICANS in patients receiving CD19 CAR-T-cells for B-cell NHL |
| 3 | 04257578 | acalabrutinib | Phase 1/2 |  | Acalabrutinib in patients receiving CD19 CAR-T-cells for B-cell NHL |

CLL: chronic lymphocytic leukemia; EAP: expanded access protocol; R/R : relapsed/refractory; NHL: non-Hodgkin Lymphoma

19 patients received CAR-T-cell therapy as part of an investigational protocol. All commercially available CD19- and BCMA-targeted CAR-T-cell products were administered at the US Food and Drug Administration (FDA)-approved dose.

**Table S2.** Demographic and clinical characteristics in all patients receiving CAR-T-cell therapy for B-cell malignancies during the study period, included and not included in the study.

| **Characteristic** | **All**  N=146^a^ | **Included**  N=72^b^ | **Not included**  N=74^c^ |
| --- | --- | --- | --- |
| Age in years, median (IQR) | 64 (56, 70) | 64 (56, 70) | 64 (56, 69) |
| Sex female, n (%) | 48 (32.9) | 31 (43.1) | 17 (23.0) |
| **Race** |  |  |  |
| Asian | 13 (8.9) | 5 (6.9) | 8 (10.8) |
| Black | 7 (4.8) | 6 (8.3) | 1 (1.4) |
| Native Hawaiian/Other Pacific Islander | 1 (0.7) | 1 (1.4) | 0 |
| American Indian/Alaska Native | 2 (1.4) | 2 (2.8) | 0 |
| White | 121 (82.9) | 58 (80.6) | 63 (85.1) |
| More than one race | 1 (0.7) | 0 | 1 (1.4) |
| **Ethnicity** |  |  |  |
| Hispanic or Latino | 11 (7.5) | 7 (9.7) | 4 (5.4) |
| Not Hispanic or Latino | 131 (89.7) | 64 (88.9) | 67 (90.5) |
| Ethnicity not reported | 4 (2.7) | 1 (1.4) | 3 (4.1) |
| **CMV serostatus** |  |  |  |
| CMV seropositive | 94 (64.4) | 72 (100.0) | 22 (29.7) |
| CMV seronegative | 51 (34.9) | 0 | 51 (68.9) |
| Unknown | 1 (0.7) | 0 | 1 (1.4) |
| **Underlying disease** |  |  |  |
| Non-Hodgkin Lymphoma | 107 (73.3) | 52 (72.2) | 55 (74.3) |
| Acute Lymphoblastic Leukemia | 11 (7.2) | 3 (4.2) | 6 (8.1) |
| Chronic Lymphocytic Leukemia | 7 (4.8) | 3 (4.2) | 4 (5.4) |
| Multiple Myeloma | 23 (15.8) | 14 (19.4) | 9 (12.2) |
| **Pre-CAR-T-cell treatments** |  |  |  |
| Prior HCT, any | 46 (31.5) | 24 (33.3) | 22 (29.7) |
| HCT within 1 year | 8 (5.5) | 4 (5.6) | 4 (5.4) |
| Years from HCT, median (IQR) | 4.2 (1.5, 6.8) | 4.2 (1.4, 5.9) | 4.3 (1.5, 7.0) |
| Prior CAR-T-cell therapy | 9 (6.2) | 4 (5.6) | 5 (6.8) |
| **CAR-T-cell target** |  |  |  |
| CD19/CD20 | 128 (84.2) | 58 (80.6) | 65 (87.8) |
| BCMA | 23 (15.8) | 14 (19.4) | 9 (12.2) |
| **CAR-T-cell product** |  |  |  |
| axicabtagene ciloleucel | 29 (19.9) | 15 (20.8) | 14 (18.9) |
| tisagenlecleucel | 4 (2.7) | 3 (4.2) | 1 (1.4) |
| lisocabtagene maraleucel | 41 (28.1) | 21 (29.2) | 20 (27.0) |
| brexucabtagene autoleucel | 26 (17.8) | 7 (9.7) | 19 (25.7) |
| idecabtagene vicleucel | 13 (8.9) | 7 (9.7) | 6 (8.1) |
| ciltacabtagene autoleucel | 10 (6.9) | 7 (9.7) | 3 (4.1) |
| investigational product | 23 (15.8) | 12 (16.7) | 11 (14.9) |

Results are reported as number with percentage in parenthesis, n (%), unless specified otherwise.

^a^146 CAR-T-cell infusion events in 141 patients; five patients received repeat infusions, three of them are included in the study.

^b^72 CAR-T-cell infusion events in 69 patients.

^c^74 CAR-T-cell infusion events in 72 patients.

**Table S3.** CMV cell-mediated immunity results in patients with and without corticosteroid use

|  | **Steroid use any**  **N=29** | **No steroids**  **N=40** | **p value^1^** |
| --- | --- | --- | --- |
| **Week 2 post-CAR-T-cells** |  |  |  |
| IE-1 [SPCs] | 18 (0–175) | 70 (6–218) | 0.13 |
| pp65 [SPCs] | 69 (0–263) | 212 (13–394) | 0.29 |
| **Week 4 post-CAR-T-cells** |  |  |  |
| IE-1 [SPCs] | 116 (8–448) | 346 (72–627) | 0.11 |
| pp65 [SPCs] | 330 (89–745) | 722 (452–912) | 0.01 |
|  | **Steroids >3 days**  **N=16** | **Steroids ≤3 days/no steroids**  **N=53** | **p value^1^** |
| **Week 2 post-CAR-T-cells** |  |  |  |
| IE-1 [SPCs] | 2 (0–33) | 59 (6–230) | 0.03 |
| pp65 [SPCs] | 61 (0–253) | 182 (14–376) | 0.18 |
| **Week 4 post-CAR-T-cells** |  |  |  |
| IE-1 [SPCs] | 84 (1–437) | 345 (69–599) | 0.08 |
| pp65 [SPCs] | 254 (1–702) | 639 (329–897) | 0.03 |

Results are in spot counts per 250000 cells (SPCs) and are reported as median (IQR).

^1^Wilcoxon rank sum test

**Table S4**: Univariate logistic regression for low week 2 CMV-CMI (IE-1 and pp65 below median)

|  | **IE-1 <median (42 SPCs)** | | **pp65 <median (155 SPCs)** | |
| --- | --- | --- | --- | --- |
| **Covariate** | **Odds Ratio (95% CI)** | **p** | **Odds Ratio (95% CI)** | **p** |
| **Sex**  Female  Male | 1.05 (0.41-2.73)  1 | 0.916 | 0.66 (0.25-1.71)  1 | 0.387 |
| **Age (continuous)** | 1.00 (0.96-1.04) | 0.947 | 0.99 (0.95-1.03) | 0.616 |
| **Prior HCT, any**  Yes  No | 0.41 (0.15-1.16)  1 | 0.092 | 1.19 (0.44-3.24)  1 | 0.734 |
| **Prior allogeneic HCT**  Yes  No | 0.23 (0.02-2.22)  1 | 0.206 | 0.23 (0.02-2.22)  1 | 0.206 |
| **Prior antitumor regimens**  (upper quartile=6)  >6  ≤6 | 0.83 (0.23-3.04)  1 | 0.782 | 2.01 (0.53-7.62)  1 | 0.305 |
| **CAR-T-cell target**  BCMA  CD19 or CD20 | 0.45 (0.12-1.66)  1 | 0.232 | 1.56 (0.44-5.48)  1 | 0.492 |
| **Absolute lymphocyte count**  **at baseline**  <300 cells/μL  ≥300 cells/μL | 3.23 (0.90-11.6)  1 | 0.072 | 1.49 (0.46-4.86)  1 | 0.511 |
| **Maximum ICANS grade**  ≥2  <2 | 10.7 (2.73-41.6)  1 | <.001 | 1.84 (0.64-5.30)  1 | 0.258 |
| **Maximum CRS grade**  ≥2  <2 | 1.05 (0.39-2.82)  1 | 0.93 | 1.35 (0.50-3.65)  1 | 0.553 |
| **Maximum CRS**  **and/or ICANS grade**  ≥2  <2 | 2.43 (0.92-6.42)  1 | 0.074 | 1.50 (0.58-3.89)  1 | 0.405 |
| **Corticosteroid use >3 days**  Yes  No | 6.60 (1.68-26.0)  1 | 0.007 | 2.01 (0.64-6.34)  1 | 0.232 |
| **IE-1 at baseline**  <median  ≥median | 3.51 (1.30-9.46)  1 | 0.013 | 1.69 (0.65-4.38)  1 | 0.281 |
| **pp65 at baseline**  <median  ≥median | 4.56 (1.66-12.6)  1 | 0.003 | 6.00 (2.12-17.0)  1 | <.001 |

**Table S5:** Univariate Cox regression analysis for CMV reactivation within 12 weeks after CARTx.

| **Covariate** | **Unadjusted HR (95% CI)** | **p** |
| --- | --- | --- |
| **Sex**  Female  Male | 0.86 (0.33–2.21)  1 | 0.75 |
| **Age (continuous)** | 0.98 (0.94–1.02) | 0.39 |
| **Prior HCT, any**  Yes  No | 0.77 (0.28–2.13)  1 | 0.62 |
| **Prior allogeneic HCT**  Yes  No | 3.21 (0.84–12.2)  1 | 0.09 |
| **Prior antitumor regimens** (upper quartile=6)  >6  ≤6 | 3.27 (1.31–8.20)  1 | 0.01 |
| **CAR-T-cell target^1^**  BCMA  CD19 or CD20 | 2.51 (0.94–6.71)  1 | 0.07 |
| **CAR-HEMATOTOX score (n=59)**  High ≥2  Low <2 | 1.11 (0.39–3.17)  1 | 0.84 |
| **Absolute lymphocyte count at baseline**  <500 cells/μL  ≥500 cells/μL | 0.53 (0.17–1.64)  1 | 0.27 |
| **Maximum CRS and/or ICANS grade (time dependent)**  ≥2  <2 | 2.17 (0.87–5.47)  1 | 0.10 |
| **Immunosuppression for CRS and/or ICANS^2^ (time dependent)**  Yes  No | 2.35 (0.92–6.0)  1 | 0.08 |
| **Corticosteroid use (time dependent)**  Yes  No | 2.53 (0.98–6.54)  1 | 0.06 |
| **Corticosteroid use for >3 days**  Yes  No | 2.63 (1.02–6.80)  1 | 0.05 |
| **Absolute lymphocyte count (time dependent)**  <500 cells/μL  ≥500 cells/μL | 0.98 (0.38–2.52)  1 | 0.97 |
| **CMV cell-mediated immunity**  **(n=69)** |  |  |
| **IE-1 at week 2**  <median (42 SPCs)  ≥median (42 SPCs) | 1.80 (0.71–4.56)  1 | 0.21 |
| **pp65 at week 2**  <median (155 SPCs)  ≥median (155 SPCs) | 3.11 (1.12–8.66)  1 | 0.03 |
| **IE-1 and/or pp65 at week 2 <median**  Yes  No | 2.83 (0.94–8.47)  1 | 0.06 |

^1^Underlying disease not shown because is represented by CAR-T-cell target (CD19/20 used for lymphoid malignancies: non-Hodgkin lymphoma, chronic lymphocytic leukemia (CLL) and acute lymphoblastic leukemia (ALL); BCMA-targeted for multiple myeloma).

^2^Immunosuppression included corticosteroids alone (n=5), tocilizumab alone (n=1) and combinations therapy (n=25) (corticosteroids and tocilizumab (n=17), corticosteroids, tocilizumab and anakinra (n=6), corticosteroids and anakinra (n=2)). Prophylactic anakinra is not shown here.

CMV-CMI, absolute lymphocyte count (ALC), CRS and/or ICANS, and immunosuppression were evaluated as time-dependent variables.

**Table S6:** Univariate Cox regression analysis for CMV reactivation after excluding participants with only 1 positive test unless that result was ≥150 IU/mL.

| **Covariate** | **Unadjusted HR (95% CI)** | **p** |
| --- | --- | --- |
| **Prior allogeneic HCT**  Yes  No | 3.20 (0.71–14.5)  1 | 0.13 |
| **Prior antitumor regimens** (upper quartile=6)  >6  ≤6 | 1.44 (0.31–6.66)  1 | 0.64 |
| **CAR-T-cell target**  BCMA  CD19 or CD20 | 1.94 (0.50–7.55)  1 | 0.34 |
| **Maximum CRS and/or ICANS grade (time dependent)**  ≥3  <3 | 1.02 (0.23–4.52)  1 | 0.98 |
| **Corticosteroid use for >3 days**  Yes  No | 3.73 (1.08–12.90)  1 | 0.04 |
| **CMV cell-mediated immunity**  **(n=69)** |  |  |
| **IE-1 at week 2**  <median (42 SPCs)  ≥median (42 SPCs) | 1.68 (0.50–5.69)  1 | 0.41 |
| **pp65 at week 2**  <median (155 SPCs)  ≥median (155 SPCs) | 1.68 (0.50–5.69)  1 | 0.41 |

**Figure S1**

**(Title)** Study protocol infographic

**(Legend)** Plasma was collected once prior to and for up to 12 weeks after CAR-T-cell infusion for PCR CMV testing; CMV cell mediated immunity (CMV-CMI) was assessed before and at week 2 and 4 after infusion. *Created with Biorender.*


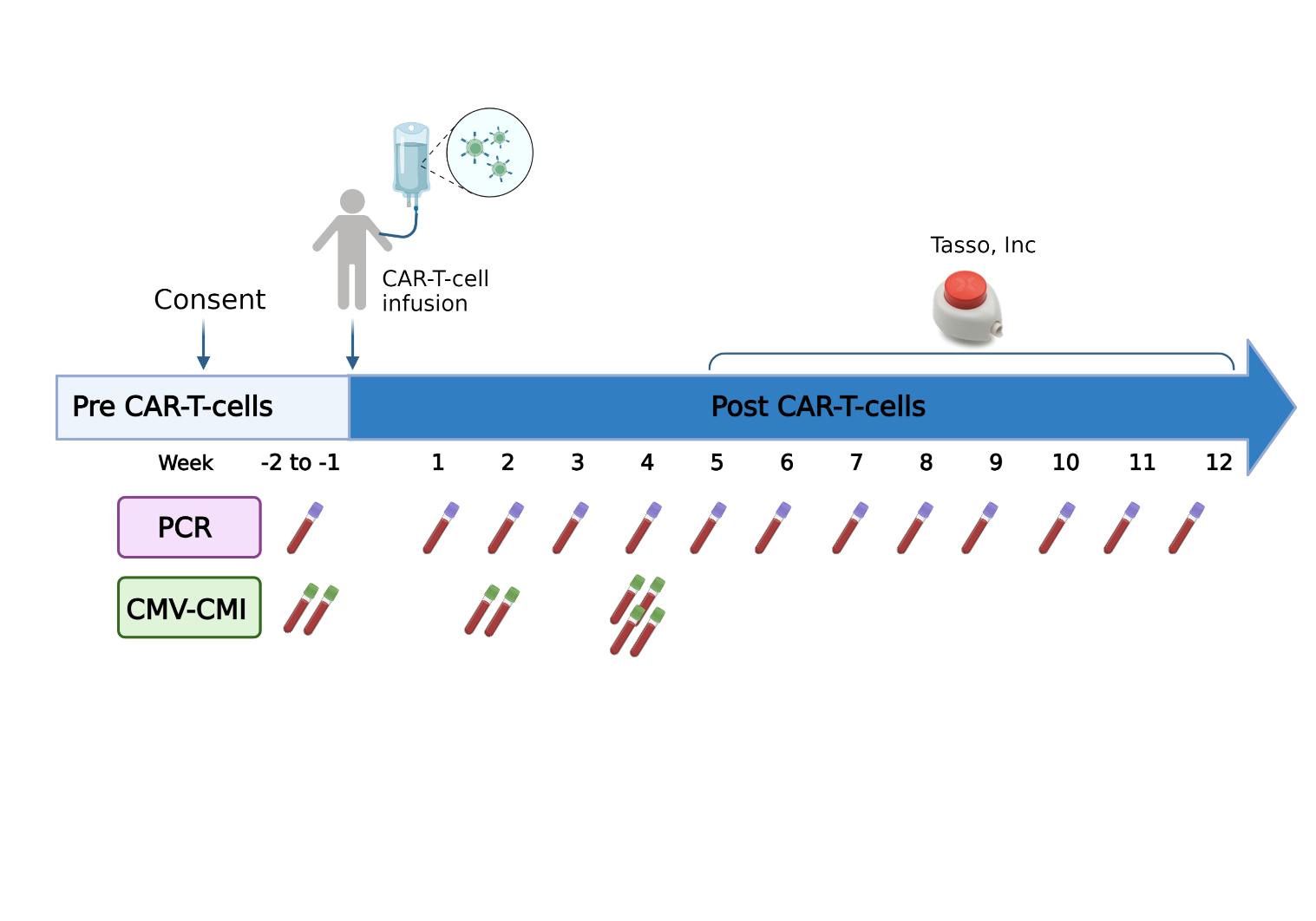


**Figure S2**

**(Title)** CMV cell-mediated immunity in CAR-T-cell therapy recipients at baseline compared to non-immunocompromised CMV-seropositive controls

**(Legend)** Box plots with T-cell responses to IE-1 and pp65 in CAR-T-cell therapy recipients at baseline and in 10 healthy controls. The horizontal line and the box represent median values and upper and lower quartiles, respectively; the circles represent all values

p=0.9 for IE-1 and p=0.28 for pp65 (Wilcoxon rank-sum test).


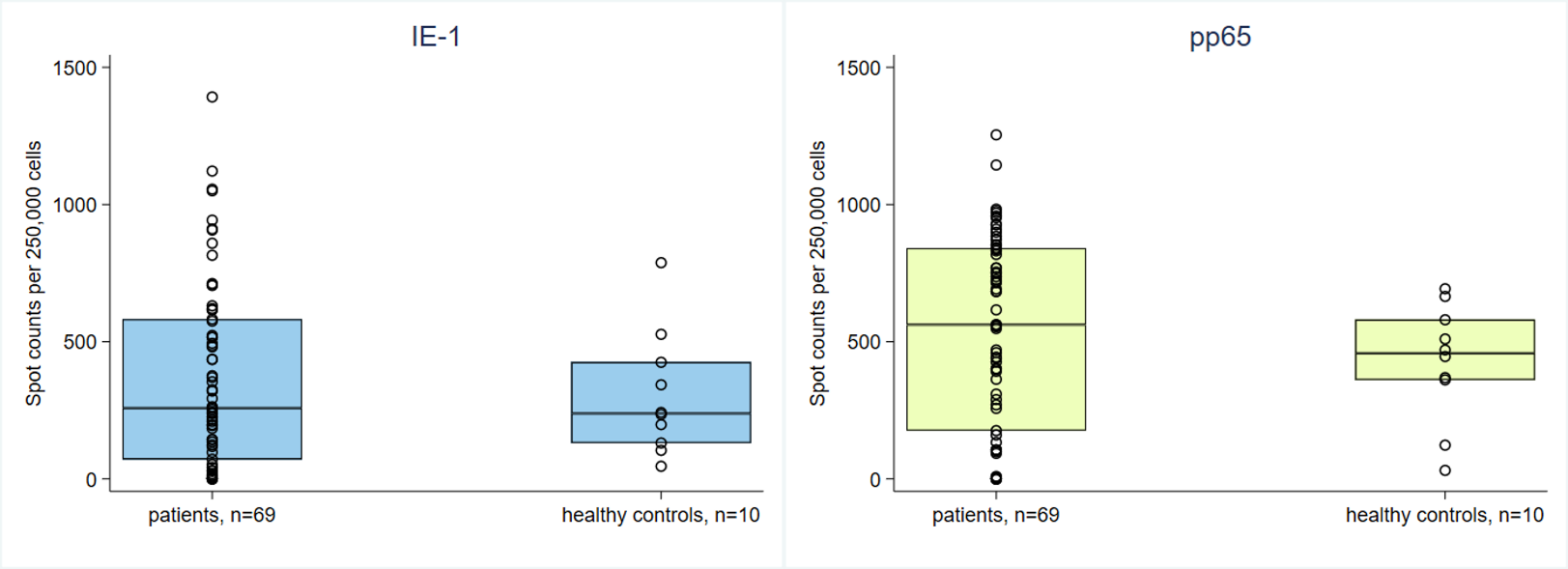


**Figure S3**

**(Title)** CMV cell-mediated immunity stratified by CAR-T-cell target

**(Legend)** CMV-specific T-cell responses to pp65 (**A.**) and IE-1 (**B.**) pre-CARTx, at week 2 and week 4 post-CARTx each timepoint in CD19/20 (red dashed line) versus BCMA (blue solid line) CARTx recipients. Lines go through the means and the vertical bars represent 95% confidence intervals.


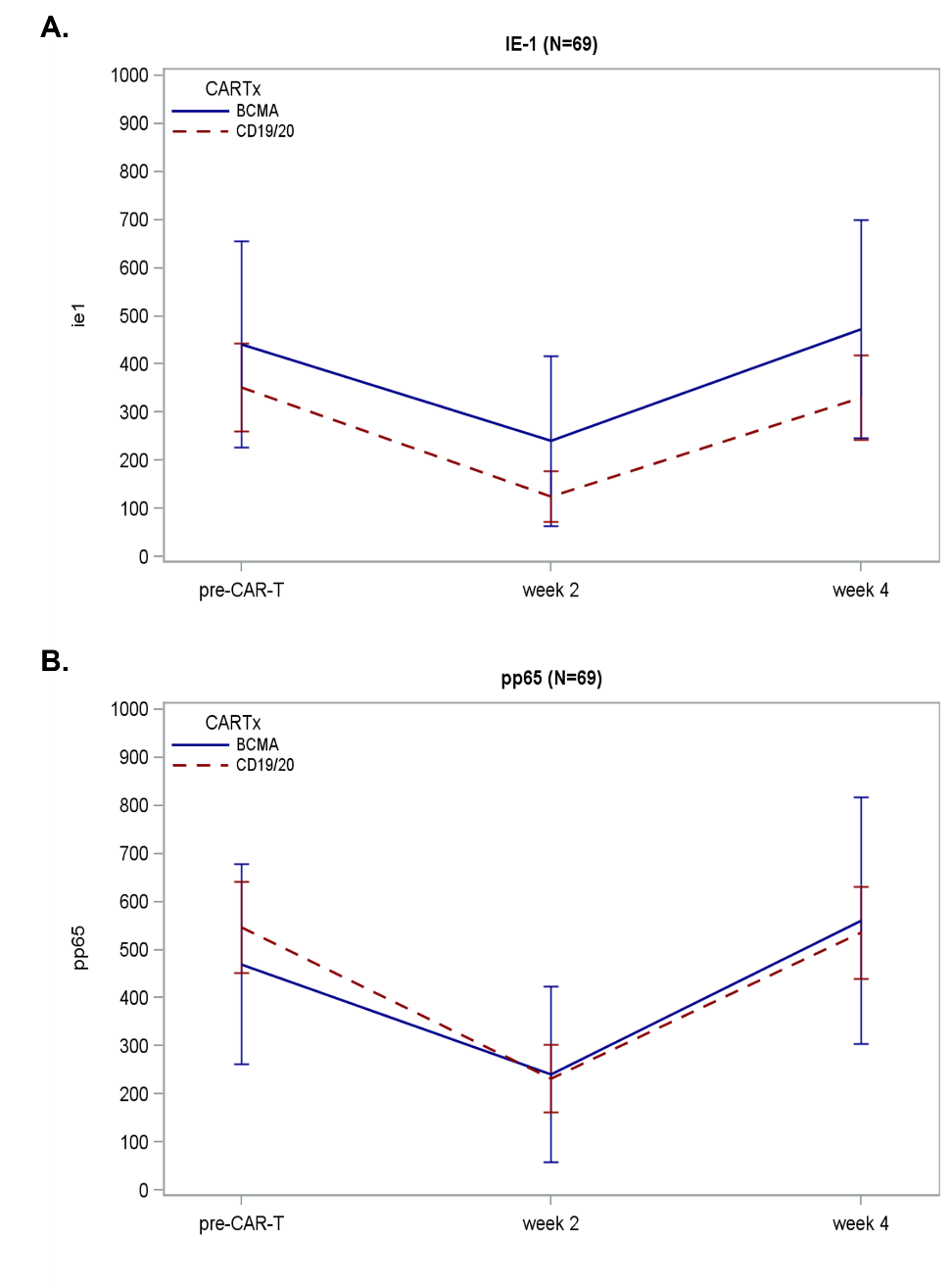


**Figure S4**

**(Title)** Receiver operating characteristic (ROC) curves of CMV-CMI at week two post-CAR-T-cell infusion as a predictor for subsequent CMV reactivation.

**(Legend)** Receiver operating characteristic (ROC) curves were computed to evaluate the performance of T-cell responses to IE-1 (**A**) and pp65 (**B**) at week two post-CAR-T-cell therapy, for predicting subsequent CMV reactivation.

Values on the plot x (y, z): x represents CMV-CMI, y, z in parenthesis are the estimated sensitivity and specificity. The red (bigger) circle highlights the optimal value according to ROC analysis. ASC indicates absolute spot counts. Median values of IE-1 (42 SPCs) and pp65 (155 SPCs) were associated with sensitivities of 61.1% and 72.2%, specificities of 52.9% and 56.9%, positive predictive values of 31.4% and 37.1% and negative predictive values of 79.4% and 85.3% respectively.


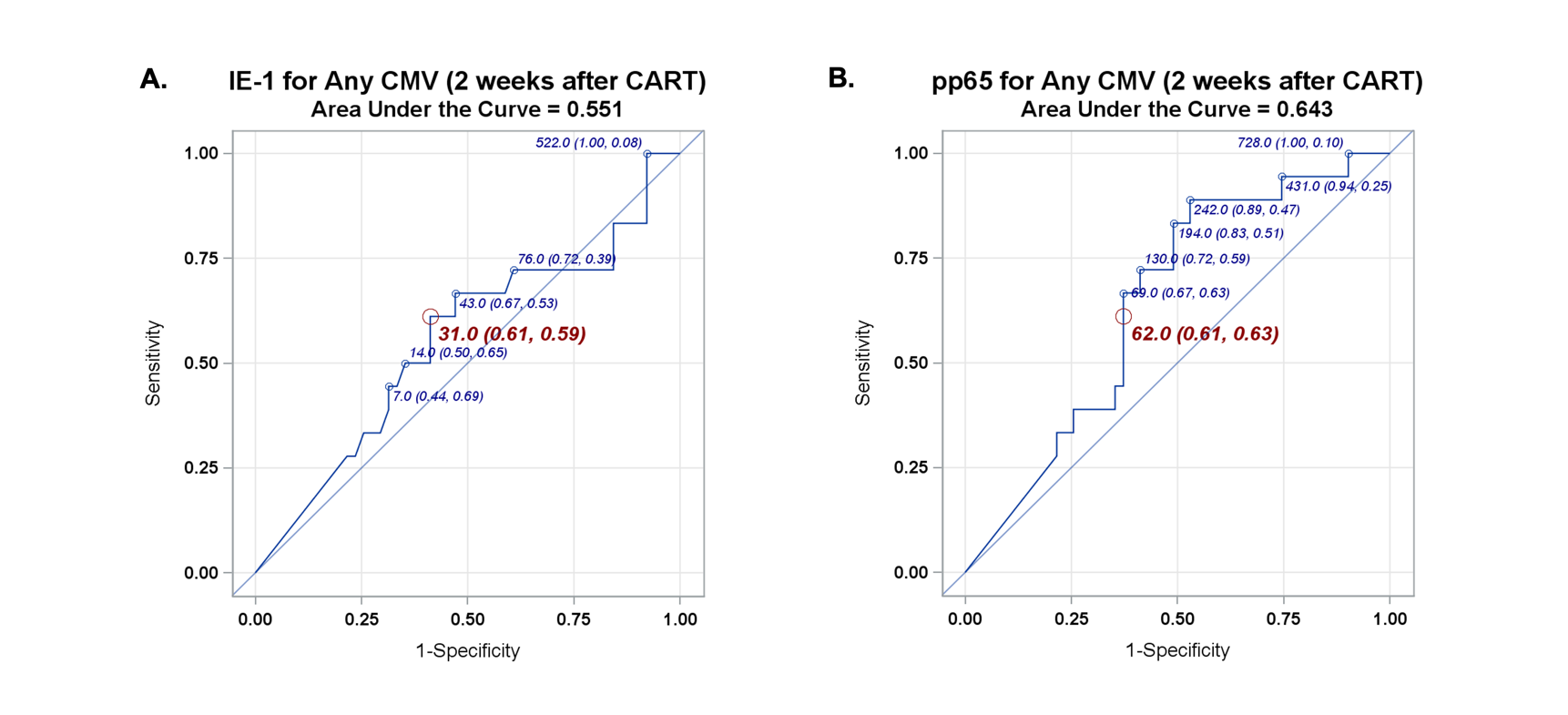


**Figure S5**

**(Title)** Absolute lymphocyte counts in patients with and without CMV reactivation

**(legend)** Box plots of absolute lymphocyte counts (ALC) in Log_10_ cells/μL at baseline and two weeks after infusion between patients with and without CMV reactivation.

The lowest value of ALC was included from day -14 to -5 (before lymphodepleting chemotherapy) for baseline values and from day 0 to week 2 for the week two time point. The bold horizontal line and the box represent median values and upper and lower quartiles, respectively; the ‘whiskers’ represent the range. The diamond shape was used for mean and points outside the range (cross and circle) are outliers.


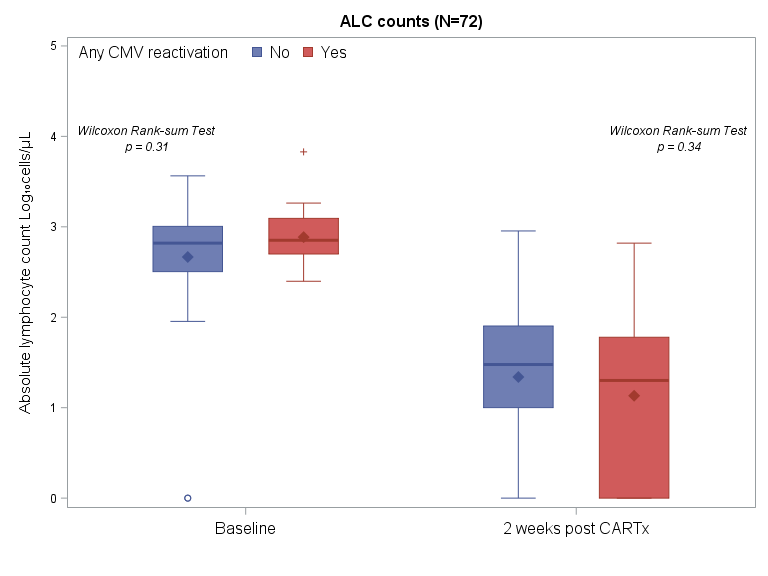


**Figure S6**

**(Title)** Cumulative incidence of CMV reactivation by week 12 post-CAR-T-cell therapy (CARTx) among participants with patients with >1 positive test or any test ≥150 IU/mL.

**(Legend):** Cumulative incidence of CMV reactivation by week 12 after excluding patients with only 1 positive test unless that result was ≥150 IU/mL, stratified by CAR-T-cell therapy target and receipt of corticosteroids. Curves start at week 2 after corticosteroid onset for CRS/ICANS. One patient who started corticosteroids on day 22 was removed, so the total number of events was N=9.


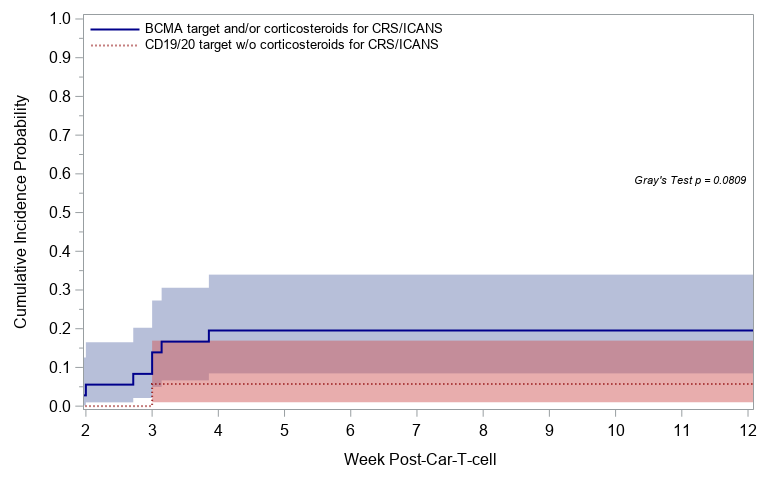


**REFERENCES**

1. Ljungman P, Boeckh M, Hirsch HH, et al. Definitions of Cytomegalovirus Infection and Disease in Transplant Patients for Use  in Clinical Trials. Clin Infect Dis **2017**; 64:87–91.

2. Li J, Liu Y, Ma L, et al. The performance of T-cell Xtend reagent in increasing blood storage times for  interferon gamma release assays. J Clin Lab Anal **2018**; 32.

3. Chemaly RF, El Haddad L, Winston DJ, et al. Cytomegalovirus (CMV) Cell-Mediated Immunity and CMV Infection After Allogeneic  Hematopoietic Cell Transplantation: The REACT Study. Clin Infect Dis **2020**; 71:2365–2374.

4. Nesher L, Shah DP, Ariza-Heredia EJ, et al. Utility of the Enzyme-Linked Immunospot Interferon-γ-Release Assay to Predict the  Risk of Cytomegalovirus Infection in Hematopoietic Cell Transplant Recipients. J Infect Dis **2016**; 213:1701–1707.

5. Turtle CJ, Hanafi L-A, Berger C, et al. Immunotherapy of non-Hodgkin’s lymphoma with a defined ratio of CD8+ and CD4+  CD19-specific chimeric antigen receptor-modified T cells. Sci Transl Med **2016**; 8:355ra116.

6. Márquez-Algaba E, Iacoboni G, Pernas B, et al. Impact of Cytomegalovirus Replication in Patients with Aggressive B Cell Lymphoma  Treated with Chimeric Antigen Receptor T Cell Therapy. Transplant Cell Ther **2022**; 28:851.e1-851.e8.

7. Chen G, Herr M, Nowak J, et al. Cytomegalovirus reactivation after CD19 CAR T-cell therapy is clinically  significant. Haematologica. 2023; 108:615–620.

8. Beyar-Katz O, Kikozashvili N, Bar On Y, et al. Characteristics and recognition of early infections in patients treated with commercial anti-CD19 CAR-T cells. Eur J Haematol **2021**; 108:1–9.

9. Solano de la Asunción C, Hernani R, Albert E, et al. Cytomegalovirus DNAemia in hematological patients undergoing CD19-directed CAR-T cell therapy: should it be systematically monitored? Clinical Microbiology and Infection **2023**; Available at: https://www.sciencedirect.com/science/article/pii/S1198743X23002343.
